# Long Term Implications of Home Healthcare Management on Mortality in Older Adults with Functional Difficulties in the Saudi Community

**DOI:** 10.3390/geriatrics6040115

**Published:** 2021-12-11

**Authors:** Khalid H. Alabbasi, Estie Kruger, Marc Tennant

**Affiliations:** 1 Ministry of Health, Jeddah 11176, Saudi Arabia; 20446883@student.uwa.edu.au; 2 International Research Collaborative—Oral Health and Equity, The University of Western Australia, Nedlands, WA 6009, Australia; marc.tennant@uwa.edu.au

**Keywords:** home healthcare, mortality, Saudi Arabia, older adult

## Abstract

Background: This study aims to investigate whether certain demographic factors of patients receiving home healthcare (HHC) interventions have any positive impact on mortality. Methods: the study included all patients who were enrolled in the HHC program in a referred medical complex, Jeddah, Saudi Arabia between the years 2017 and 2020 (593 patients). Results: A total of 6548 HHC visits were received during the study period. From the total number of visits, 3592 (54.9%) HHC visits were scheduled in the year 2020 compared to 157 (2.4%) scheduled HHC visits in 2017 (*p* < 0.001). The most successful HHC visits were provided in 2020 compared with the year 2017 (2193 vs. 132; *p* < 0.001). The cancelled HHC visits were observed to be the lowest (194) in 2019. Three explanatory variables of mortality [age, having a major diagnosis (diabetes mellitus, cerebrovascular diseases, and bedridden), and having more cancelled visits] made a statistically significant contribution to the logistic regression model after controlling for other variables. Suffering from cerebrovascular diseases and/or bedridden were the strongest predictor of death in patients receiving HHC. Conclusions: During the 2020 pandemic, there was a sharp increase in HHC compared to previous years. Three significant explanatory variables of mortality [age, having a major diagnosis (diabetes mellitus, cerebrovascular diseases, and bedridden), and having more cancelled visits] were reported.

## 1. Introduction

As the population ages, the prevalence of functional disability is expected to increase due to age-related chronic or debilitating conditions, particularly among the oldest people [1]. Functional difficulties (as a marker of frailty) have been significantly associated with increased risk of falling, reliance on personal assistance, and high healthcare service utilisation and costs [2,3,4].

Saudi Arabia is currently entering uncharted territory in relation to the proportion of its aging population. Recent data show that there has been a significant change in Saudi Arabia’s age structure owing to the fall in birth rates of the Saudi population and the increase in life expectancy. In predicting continued growth of an older population, the proportion of Saudi people ages 65 and above is projected to be nearly 18.4% of the total population of 40 million by 2050 [5]. Furthermore, prevalence rates of chronic diseases are increasing in Saudi Arabia, contributing almost 60% of all premature deaths in 2016, estimated to rise to 73% by 2020 [6]. Of these, diabetes and hypertension are the most common, with prevalence rates of 23% and 26% respectively [6]. These phenomena will trigger several concerns and challenges to maintain healthcare demands in term of quantity, quality, and type of healthcare services and facilities; and thus, reviewing the number of health support services for elders is timely. The Saudi Ministry of Health (MOH) has been engaged in widespread efforts to expand health care through establishing a publicly funded home healthcare (HHC) program in 2009, which is administered by regional health authorities [7]. The HHC program is one part of a continuum of health care that allows elderly patients to receive care in their own homes as opposed to the hospital care setting, assuming that this program will optimise care management, reduce hospitalisation, and also reduce the risk to any hospital-acquired infection. The home healthcare environment in Saudi Arabia involves less direct physician contact and more professional nurse-led teams, and the physician relies to a greater degree on the nurse to make assessments and communicate findings [8].

More information concerning home healthcare service utilisation is needed to meet the needs of the rapidly aging population in Saudi Arabia. Our current literature search showed no data available or study regarding the assessment of the HHC program among the Saudi population. Presently, this study aims to investigate whether certain demographic factor of patients receiving home healthcare interventions have any positive impact on mortality. It was hypothesised that patients receiving higher successful home health care visits would be less likely to die.

## 2. Materials and Methods

### 2.1. Home Healthcare Program Description

This program comprises a set of health activities and services provided to patients at their homes, in compliance with certain standards and mechanisms applied by a work team qualified and prepared for this very purpose [7,8]. The program takes as its goal the provision of healthcare for patients at their homes safely and comfortably, without the need for hospitalisation, in addition to helping patients with recovery and restoring their health, in terms of the physical, mental, rehabilitative, and social aspects [7]. It also protects patients from contracting infections while staying at the hospital for long periods and makes it easier for the patient’s family and relatives to visit them.

The program includes a comprehensive physical and vital signs assessment, medical history reviews, reviewed disease knowledge and medications adherence, lifestyle habits, and social services/support. This program is necessary for raising people’s awareness and informing them of the proper health instructions to be followed by patients and their families [7,8]. This role is aptly incurred by the health team during the delivery of the health service. The program also provides the necessary medical equipment for patients, in coordination with the relevant governmental and private health sectors [7]. Finally, the program is effective in the rationalisation of expenditure at hospitals, by lowering the proportion of hospitalisation, and reducing the frequency of visits to hospitals, especially by patients with chronic and aging-associated diseases.

The MOH made it clear that, in order for the Home Healthcare Program to be carried out, there are certain criteria and requirements to be met by patients. A patient (aged 18 years and above) qualified to receive home healthcare services needs to fulfil the following criteria: (a) a transfer request to the HHC program should be prescribed by the physician, (b) the patient has to be in one of the groups targeted by the program, (c) the patient’s health situation is supposed to be relatively stable, (d) the patient should be living within a radius of 50 km from the hospital (up to 30 min by car), (e) the transfer must be approved by the head of the family (homeowner) so as to be followed up by the health team, (f) the appropriateness and safety of a curative environment at home, and (g) the availability of a caregiver (family member) to take care of the patient.

### 2.2. Study Design, Setting and Sample Selection

This was an analytical, retrospective study that included all patients who were enrolled in the HHC program in a referred medical complex, Jeddah, Saudi Arabia between the years 2017 and 2020. The program started with one field team specialised in home healthcare in 2017, and this increased to four teams in 2020.

### 2.3. Measures

Patients enrolled in the HHC program are followed up until their condition has been resolved, they have been transferred to another program, or they have passed away. The patients’ visitation records are linked to the hospital “InterSystems *TrakCare HIS*”. Data were obtained directly from the hospital *TrakCare* system, which contained the home care visits information. Patients who received at least one in-home care visit were eligible to be included in this study. Visiting data included date of first scheduled visit, number of successful visits, and cancelled visits for all patients enrolled in the HHC program. Mortality/survival (as an outcome of interest) was also obtained from the system. Demographic data (e.g., age, gender, major diagnosis) were collected and served as the principal descriptive components of the study. All data identifying beneficiaries, physicians, and institutions were encrypted to ensure privacy.

### 2.4. Ethical Approval

Before carrying out the research, ethics application was submitted to the Ministry of Health in Saudi Arabia (KSA:H-02-J-002), and the Human Research Ethics Committee at the University of Western Australia (RA/4/20/6317). The proposal was reviewed, and approval was granted to continue with the research. The extracted data were then coded, to ensure the confidentiality and privacy of all patients. The dataset was saved in a password protected computer, only e accessible by the research team.

### 2.5. Data Analysis

For analysing the data, the ‘Statistical Package for Social Sciences’ (SPSS Inc., Chicago, IL, USA) version 21 was used. The data were first screened for the presence of any entry errors and outliers. Descriptive statistics were undertaken and categorical data (e.g., gender and patients’ health and diseases) were reported as frequency and percentage (%) and continuous variables, e.g., age and number of visits as a median and interquartile range (IQR). For inferential analysis, the bivariate analysis chi-square test and Mann-Whitney tests were run to explore any statistically significant association between explanatory categorical and continuous variables with the dependent variable death outcome ‘No’ or ‘Yes’.

The binary logistic regression was run to underscore the potential significant (*p* < 0.05) mortality (death) among patients enrolled in the HHC program accounting for age and other factors. The variable selection from the Chi-square and Mann-Whitney test into the binary regression was based on the statistical significance (*p* < 0.05).

## 3. Results

Between 2017 and 2020, a total of 593 beneficiaries were enrolled in the HHC program at a referred medical complex in Jeddah, Saudi Arabia. There were 228 males (38.4%) and 365 females (61.6%) with a median age of 78 years (interquartile range, 60–96 years) in the study group. When stratified by age, the highest home healthcare service utilisation was in those aged 75–84 years (23.9%) and the lowest utilisation was in those aged ≤55 years (4.4%). Females were more likely to receive home healthcare visits than males (62% vs. 38%). The gender difference increased with age (Table 1). Considering the total HHC visits, individuals aged 75–84 years accounted for the greatest proportion of total HHC visits (34.3%), while those ≤55 years of age represented the lowest proportion of visits (11.4%).

**Figure 1:**
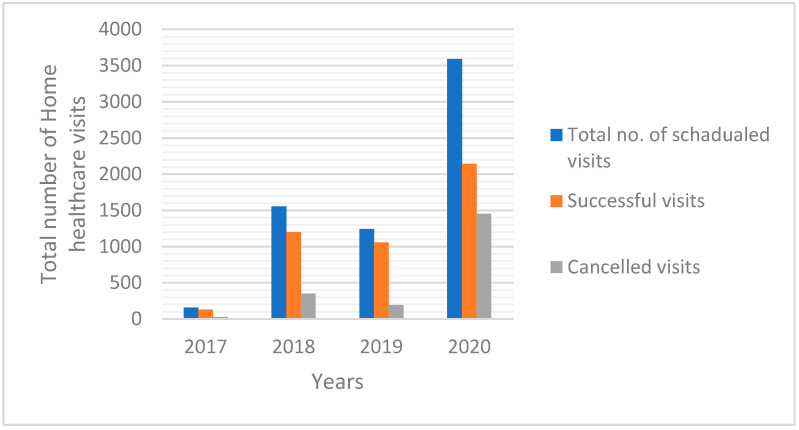
Frequency distribution for home healthcare visits (successful visits vs. cancelled visits) overtime (2017–2020), *p* = 0.001.

A total of 6548 HHC visits were received during the study period. From the total number of visits, 3592 (54.9%) home healthcare visits were scheduled in the year 2020 compared to 157 (2.4%) scheduled HHC visits in 2017 (*p* < 0.001). Of these, the highest successful HHC visits were provided in 2020 compared with the year 2017 (2193 vs. 132; *p* < 0.001). The cancelled HHC visits were observed to be the lowest (194) in 2019 (Figure 1). A lower figure was observed in 2019 compared to 2018, as a result of MOH launching a separate home healthcare program in the rural area, whereabouts those residing there were transferred to them (Figure 1).

According to the ICD-10-CM coding system, the four major diagnoses for patients receiving HHC visits were hypertension (*n* = 450, 75.9%), cerebrovascular diseases (CVD, *n* = 393, 66.3%), diabetes mellitus (*n* = 339, 57.2), and bedridden (*n* = 158, 26.6%; Table 2). Table 2 shows the characteristics of patients who had received HHC visits alongside the bivariate analysis of factors associated with mortality.

As shown in Table 3, three explanatory variables of mortality [age, having a major diagnosis (diabetes mellitus, cerebrovascular diseases and bedridden), and having more cancelled visits] made a statistically significant contribution to the logistic regression model after controlling for other variables. Suffering from cerebrovascular diseases and/or bedridden were the strongest predictors of death in patients receiving HHC. Patients who suffered from cerebrovascular diseases were 3.11 (1.72–5.63, *p* ≤ 0.001) times more likely to die when receiving home healthcare than those patients who did not have cerebrovascular diseases. Furthermore, bedridden patients are at higher risk of death (AOR = 2.91; 95%CI = 1.78–4.76, *p* ≤ 0.001) when receiving care at home than those who were not bedridden. Patients who cancelled their visits more frequently were also significantly more likely to be at higher risk of mortality (OR = 0.89, 95%CI = 0.83–0.97, *p* ≤ 0.001) than those who had fewer cancelled HHC visits.

## 4. Discussion

In this cross-sectional, retrospective study the relationship of demographic factors of patients receiving home healthcare interventions, with mortality (alongside functional difficulties) in older adults was investigated. There is a considerable body of literature focusing on the age and gender differences in healthcare utilisation [9,10]. Therefore, exploration of age and gender differences in health behaviours is an important step toward an understanding of future practice and education needs. Females and patients aged 75 to 84 years were more likely to receive home healthcare visits, than men or those aged ≤55 years. Perhaps women are taking more responsibility for their health, potentially related to risk perception, than men [11,12]. Additionally, individuals aged over 75 years accounted for a higher proportion of HHC visits compared to those younger than 55 years. This is probably because people over 75 suffer from more diseases/medical conditions than younger people [13]. Thus, HHC may in fact also be cost effective as a preventative measure against the potential expenses associated with hospital admission in older adults.

It has been observed in our study that the number of scheduled and successful home healthcare visits were higher in the year 2020 compared with the previous years. This might be credited to the unprecedented and stringent measures taken by the Saudi Ministry of Health in response to the COVID-19 outbreak, including the provision of more financial support to home health providers, expanding provider licensures to certify use of home health, and facilitating wider use of telehealth [14]. Globally, the COVID-19 pandemic has upended the entire healthcare delivery system, including home healthcare. Yet, the full impact of the pandemic on the home healthcare visit system and outcome has received little attention. To better understand these effects, evidence-based analysis should be examined.

Hypertension, cerebrovascular diseases, diabetes and being bedridden were the principal diagnoses of home health care recipients. This is of particular concern given the growing number of diagnosed diabetes and CVD cases in the Saudi community, specifically among the elderly. It has been predicted that the prevalence of type 2 diabetes will reach 40% of the total Saudi population in 2025 and 45% in the year 2030 [15]. Even though it is estimated that 90% of lifestyle-related disease and illness, including heart disease and type 2 diabetes are largely preventable, they remain the greatest cause of mortality among aging society in most countries [16].

For the study hypothesis, being old, having a major chronic condition and having more cancelled visits were predictive factors of death among this study group. Suffering from CVD was the most important risk indicator of mortality, and could be related to the fact that, in comparison to the hospitalised patient, patient in home health care often has a greater role in determining how and even if certain interventions will be implemented. For example, they may choose to take the medication at irregular times, despite advice about the importance of a regular medication schedule. It is interesting to note that reading skill, cognitive ability, and financial resources were reported to have an impact on the ability of home health care patients to safely manage their medication regimens [17].

Furthermore, bedridden patients were at higher risk of death when receiving care at home than those who were not bedridden. Most home-bound patients receive assistance from family members or other informal caregivers. Therefore, professional clinicians have no authority over these caregivers. Further, the home environment and the intermittent nature of professional home health care services may limit the clinician’s ability to observe the quality of care that informal caregivers deliver—unlike in the hospital, where care given by support staff may be more easily observed and evaluated. It is essential for HHC providers to improve health education of patients and their family, pay more attention to follow up after discharge and provide for care needs at home.

Moreover, the number of successful visits were expected to be associated with lower mortality rates. In the present study successful visits lost significant association with death when modelled with other predictive factors and this reflects the prominence of the number of cancelled visits over and above the number of successful visits. In most cases, visit cancellation was caused by either relocation of a patient’s residence, declining phone calls, or unavailability of caregivers. To the best of our knowledge, these findings have never been reported elsewhere.

A few limitations should be acknowledged when interpreting the findings. First, all participants were recruited from an HHC program in Jeddah, and as such the findings are not generalisable to all patients enrolled in the HHC program in Saudi. Second, the claims data did not provide detailed demographic and socio-economic data, or medical backgrounds of the beneficiaries, which precluded analysis of possible contributing factors such as education level, economic background, caregiver status, family composition, polypharmacy, and inappropriate prescription for home healthcare utilisation. These and other factors, including the impact of the COVID-19 pandemic on home healthcare, need to be considered.

## 5. Conclusions

Females were more likely to receive home healthcare visits than males and the gender difference increased with age. Three significant explanatory variables of mortality [age, having a major diagnosis (diabetes mellitus, cerebrovascular diseases, and bedridden), and having more cancelled visits] were reported. During the 2020 pandemic, there was a sharp increase in HHC compared to previous years. The study model has indicated that those who cancelled their visits more frequently had a higher risk of mortality. Further qualitative studies should be conducted to examine the reasons of cancelled HHC appointments. Barriers to home healthcare visits need to be addressed, and initiatives and strategies to increase successful visitation practices should be implemented.

## Figures and Tables

**Table 1:** Age–sex prevalence of patients receiving home healthcare services between 2017–2020.

No. of Home Healthcare Patients * (% of Beneficiaries)				No. of Home Health Care Visits (%)			Home Healthcare Visits per Patient Median (IQ) *	
Age (Year)	Males	Females	Total, *n* (%)	Males	Females	Total, *n* (%)	Males	Females
18–55	49 (8.3)	26 (4.4)	75 (12.6)	576 (8.8)	171 (2.6)	747 (11.4)	9.0 (16.5)	3.5(2.75)
56–74	79 (13.3)	85 (14.3)	164 (27.7)	750 (11.4)	895 (13.2)	1645 (25.1)	6 (12)	7.0(16)
75–84	47 (7.9)	142 (23.9)	189 (31.9)	641 (9.8)	1604 (24.5)	2245 (34.3)	10 (18)	9.0(12.5)
+85	53 (8.9)	112 (18.9)	165 (27.8)	515 (7.9)	1396 (21.6)	1911 (29.1)	8 (10.5)	9.0(15)
Total	228 (38.4)	365 (61.6)	593 (100)	2482 (38)	4066 (62)	6548 (100)		

* *p* < 0.001, between males and females in the columns.

**Table 2:** Total Home Healthcare patients’ characteristics over 4 years 2017–2020 and bivariate analysis for factors associated with home death, 4-years analysis (2017–2020), Saudi (*n* = 593).

Characteristics		F (%)/Median (IQR)			*p*-Value
		Total	Home Death		
			No	Yes	
**Home healthcare patients ^a^**		593 (100)	450	143	0.001
**Age** median (IQR) ^b^		78 (18)	77 (21)	80 (15)	0.001
**Gender ^a^**	**Males**	228 (38.4)	277 (75.9)	88 (24.1)	0.54
	**Females**	365 (61.6)	173 (75.9)	55 (24.1)	
**Patient’s major diseases ^a^**	**Hypertension**	450 (75.9)	321 (71.3)	129 (28.7)	0.001
	**Diabetes**	339 (57.2)	275 (68.9)	124 (31.1)	0.001
	**Cerebrovascular diseases**	393 (66.3)	266 (67.7)	127 (32.3)	0.001
	**Bedridden**	158 (26.6)	94 (59.5)	64 (40.5)	0.001
	**Others**	45 (7.6)	159 (84.1)	30 (15.9)	0.001
**Successful visits** median (IQR) ^b^		5 (9)	6 (10)	4 (6)	0.001
**Cancelled visits** median (IQR) ^b^		2 (5)	1 (2)	2 (5)	0.001

^a^ Chi-square test and ^b^ Mann-Whitney U test were used for comparisons.

**Table 3:** Binary logistic regression predicting the likelihood of home death among patients enrolled in home healthcare program, 4-year analysis, Saudi (*n* = 593).

Explanatory Variables		B	Wald	OR (95%CI)	*p*-Value
**Age**		0.02	5.095	1.02 (1.00–1.04)	0.024
**Patient’s major disease**					
**Hypertension**	**No**			Reference	
	**Yes**	0.496	1.990	1.64 (0.82–3.27)	0.158
**Diabetes**	**No**			Reference	
	**Yes**	0.841	7.422	2.32 (1.27–4.25)	0.006
**Cerebrovascular diseases**	**No**			Reference	
	**Yes**	1.135	14.046	3.11 (1.72–5.63)	0.001
**Bedridden**	**No**			Reference	
	**Yes**	1.067	18.184	2.91 (1.78–4.76)	0.001
**Others**	**No**			Reference	
	**Yes**	−0.118	0.178	0.89 (0.51–1.54)	0.673
**Successful visits**		−0.034	3.805	0.97 (0.94–1.00)	0.051
**Cancelled visits**		0.112	8.223	0.89 (0.83–0.97)	0.004

## Data Availability

The datasets analysed during this study are available from the corresponding author upon reasonable request.
